# Dynamic recruitment of UFM1-specific peptidase 2 to the DNA double-strand breaks regulated by WIP1

**DOI:** 10.1007/s42764-022-00076-z

**Published:** 2022-08-10

**Authors:** Bo Qin, Jia Yu, Fei Zhao, Jinzhou Huang, Qin Zhou, Zhenkun Lou

**Affiliations:** 1 Department of Oncology, Mayo Clinic, Rochester, MN 55905 USA; 2 Division of Clinical Pharmacology, Department of Molecular Pharmacology and Experimental Therapeutics, Mayo Clinic, Rochester, MN 55905 USA

**Keywords:** UFSP2, Ufmylation, ATM, WIP1

## Abstract

The ufmylation ligase-UFL1 promotes ATM activation by monoufmylating H4 at K31 in a positive-feedback loop after double-strand breaks (DSB) occur, whereas UFM1 Specific Peptidase 2 (UfSP2) suppresses ATM activation, but the mechanism of recruitment of UfSP2 to the DSB finetuning DNA damage response is still not clear. Here, we report that UfSP2 foci formation is delayed compared to UFL1 foci formation following the radiation insult. Mechanistically, UfSP2 binds to the MRN complex in absence of DSB. Irradiation-induced phosphorylation of UfSP2 by ATM leads to the dissociation of UfSP2 from the MRN complex. This phosphorylation can be removed by the phosphatase WIP1, thereby UfSP2 is recruited to the DSBs, deufmylating H4 and suppressing ATM activation. In summary, we identify a mechanism of delicately negative modulation of ATM activation by UfSP2 and rewires ATM activation pathways.

## Introduction

DNA damage is an alteration in the chemical structure of DNA, and it insults genomic stability. Double-strand break (DSB) is the most lethal type of DNA damage. Once DNA damage happens, DNA damage response signaling is swiftly launched. Following induction of DSBs, the apical kinase ATM is activated and phosphorylates H2AX at Ser139 (Lavin, 2008; Lee & Paull, 2004; Sun et al., 2005; Uziel et al., 2003). Phosphorylated H2AX recruits MDC1 protein, which serves as a docking platform for recruiting other DDR proteins to the DSBs (Burma et al., 2001; Goldberg et al., 2003; Jungmichel et al., 2012; Liu et al., 2012; Lou et al., 2003; Stewart et al., 2003). Failure of precise DNA damage response and repair causes many diseases, for example, cancer and neurodegenerative disorders (Kastan et al., 2001; Lavin & Shiloh, 1997).

ATM activation could be regulated by multiple post-translational modifications, such as ubiquitination, phosphorylation, acetylation, and methylation, which are discovered to regulate the DNA damage signaling (Van & Santos, 2018). The methyltransferase Suv39h1 trimethylates Histone H3 at lysine 9, and recruits Tip60 to DSBs (Ayrapetov et al., 2014). Tip60 acetylates ATM, inducing ATM autophosphorylation and activation (Sun et al., 2009). Furthermore, the MRN complex is another critical activator for ATM (Andegeko et al., 2001; Bekker-Jensen et al., 2006; Falck et al., 2005; Lee & Paull, 2004, 2005). Its subunit NBS1 can be K63 linkage polyubiquitinated by the E3 ligase SKP2 and this ubiquitination enhances NBS1 interaction with ATM, leading to recruitment of ATM to the DSBs, where ATM is activated (Wu et al., 2012).

Ufmylation is a recently identified protein modification. Similar to the ubiquitination system, conjugation of UFM1 protein to the substrate is mediated by a three-step enzymatic cascade—E1, E2, and E3 (Komatsu et al., 2004b; Tatsumi et al., 2010). Ubiquitin-like modifier activating enzyme 5 (UBA5) works as the E1 enzyme in the ufmylation system and activates the C-terminal carboxylate group of UFM1 via an adenylate intermediate to form UBA5-Cys-UFM1 thioester to catalyze the transfer of UFM1 to E2 protein-UFM1 conjugating enzyme 1 (UFC1) through a transthiolation reaction (Komatsu et al., 2004a). The resultant UFC1-Cys-UFM1 thioester transfer the UFM1 to its substrates with the catalysis of E3 UFM1-specific ligase 1 (UFL1), forming a covalent bond between lysine and C-terminal carboxylate group of UFM1 (Tatsumi et al., 2010). Accordingly, UFM1-specific protease UFSP1 and UfSP2 process UFM1 C-terminal sequences for activation and remove the UFM1. In human, only UfSP2 is functional (Kang et al., 2007).

The UFM1 conjugation system is very conservative from plants to metazoan. *Uba5* Knockout mice studies suggest a critical role in the erythrocyte differentiation (Tatsumi et al., 2011). Consistently, UFL1 and UFM1 knockout mice also show erythropoiesis defects during development. The UFM1 conjugation system also affects ER stress and the fatty acid metabolism (Cai et al., 2015; Lemaire et al., 2011; Tatsumi et al., 2011; Yoo et al., 2014). In addition, UfSP2 plays a role in GPCR biogenesis. Recently, the biological functions of the UFM1 conjugation system attract increased attention. Many substrates have been identified, such as UFBP1, ASC1, MRE11, histone H4, etc. (Cai et al., 2015; Qin et al., 2019; Yoo et al., 2014).

Previously we and the Xu lab reported that the ufmylation system regulates ATM activation, DNA damage response, and DNA repair (Qin et al., 2019; Wang et al., 2019). The E3 ligase-UFL1 monoufmylates H4 at K31, which is recognized by STK38 (Qin et al., 2019, 2020). Then, STK38 recruits Suv39h1 to DSB sites and trimethylates H3K9 (Qin et al., 2020). Tip60 recognizes this trimethylation and acetylates ATM, inducing ATM autophosphorylation and activation (Qin et al., 2019). As a positive-feedback loop, ATM further phosphorylates UFL1 at Ser462, and amplifies the ATM activation signaling. In addition, UFL1 can ufmylate MRE11 to promote ATM activation (Wang et al., 2019). To prevent excessive DNA damage response-induced cell apoptosis, cells need to fine-tune the amplitude of ATM activity to govern the severity of DNA damage (Qin et al., 2019). However, the mechanism, especially negative regulation of the ufmylation-mediated ATM activation, is still unclear. Here, we show that UfSP2 binds to the MRN complex in the unstressed cells. Following irradiation, UfSP2 is phosphorylated by ATM and dissociated from the MRN complex. This phosphorylation can be removed by the phosphatase WIP1, resulting in UfSP2 binding to the MRN complex to fine-tune ATM activity.

## Results

### UfSP2 suppresses ATM activation through deufmylation of H4K31

**Fig. 1:**
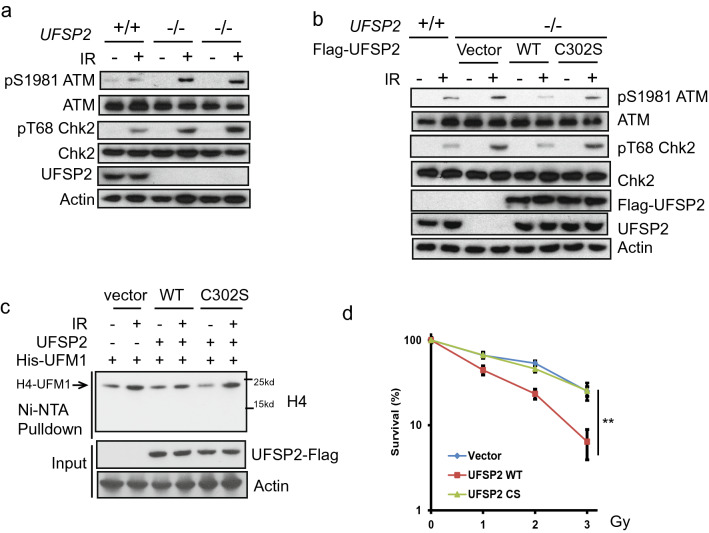
UfSP2 suppresses ATM activation. **a** Parental and two different Cripsr-cas9 mediated UfSP2 knockout 293T cells were then treated with or without 0.5 Gy IR. Cell lysates were incubated with indicated antibodies. **b** UfSP2 knockout 293 T cells expressing Vector, wildtype UfSP2, or catalytic dead mutant and parental cells were irradiated. **c** Detection of ufmylated H4 in the UfSP2 knockout 293 T cells expressing Vector, wild-type UfSP2, or catalytic dead mutant with His-UFM1 plasmids. **d** Survival curve of UfSP2 knockout cells expressing vector, wildtype UfSP2, or catalytic dead mutant treated with indicated doses of IR

UfSP2 is indicated in the suppression of ATM activation (Qin et al., 2019; Wang et al., 2019). To explore the underlying mechanism, we generated UfSP2 knockout out cells with CRISPR-Cas9 technique. Depletion of UfSP2 enhanced the phosphorylation of ATM Ser1981, which is the marker for ATM activation (Fig. 1a). To further test that the deufmylase activity of UfSP2 is important for ATM inactivation, we reintroduced the wildtype (WT) and C302S UfSP2, which loses the deufmylase activity, into UfSP2-deficient cells and found that restoration of WT UfSP2, not the C302S mutant, impaired ATM activation (Fig. 1b). Previously, we discovered that UFL1 is responsible for H4K31 monoufmylation following IR and induces ATM activation (Qin et al., 2019). To test the effect of UfSP2 on H4K31 monoufmylation, we transfected WT UfSP2 and C302S UfSP2 with His-UFM1 into UfSP2-deficient cells and treated the cells with IR. We found that expression of WT UfSP2, but not the C302S mutant, suppressed H4K31 monoufmylation (Fig. 1c). Consistent with compromised ATM signaling, the UfSP2 knockout cells expressing WT UfSP2 were more sensitive to IR compared to UfSP2-deficient cells, whereas the cells expressing C302S displayed similar sensitivity to UfSP2-deficient cells as shown in the clonogenic survival assays (Fig. 1d). These results suggest that UfSP2 deufmylates histone H4 and blocks ATM activation.

### Dynamics of UfSP2 and UFL1 foci formation following IR

**Fig. 2:**
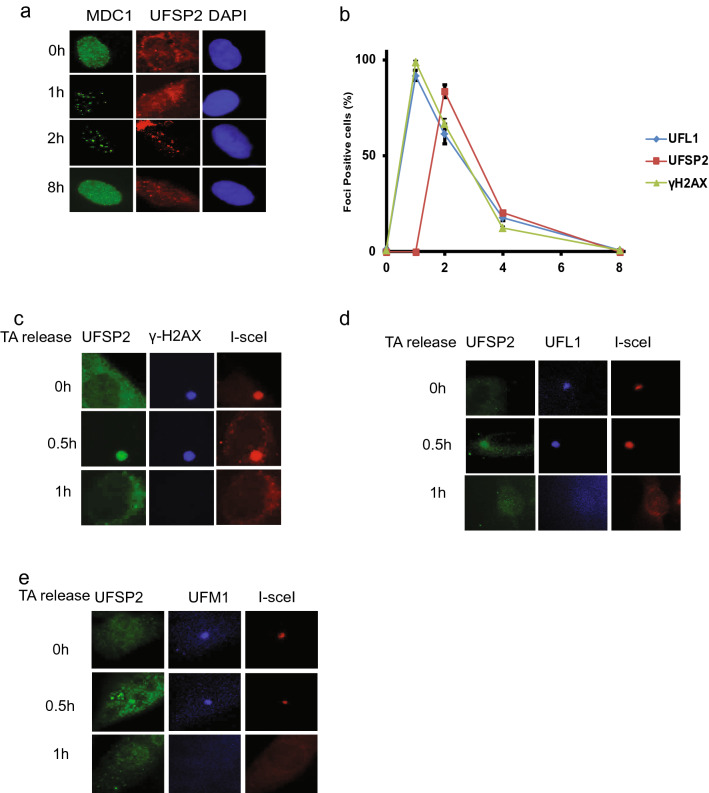
Recruitment of UfSP2 to DSB is later than UFL1 recruitment following IR. **a** Representative image of MDC1 and UfSP2 foci formation at indicated time points after IR. **b** Quantification of MDC1, UfSP2, and γH2AX foci positive cells. **c**–**e** Triamcinolone acetonide (TA) induces sing DSB by the translocation of RFP-I–SceI–GR fusion protein from the cytoplasm to the nucleus. Proteins were detected by indicated antibodies (**c** UfSP2 and γH2AX antibodies, **d** UfSP2 and UFL1 antibodies, and **e** UfSP2 and UFM1 antibodies) at different time points after TA washout

Our previous studies showed that UFL1 is recruited to DSB to ufmylate H4K31 (Qin et al., 2019). As UfSP2 removes UFM1 from its substrate, so we hypothesized that UfSP2 could be recruited DSBs to remove ufmylated substrate. To test this hypothesis, we monitored the localization of UfSP2 following irradiation and found that UfSP2 foci could be detected at 2 h following IR (Fig. 2a). To study the dynamics of foci formation, we monitored UfSP2, UFL1, and γH2AX foci at different time points after IR and found that UFL1 foci positive cells and γH2AX foci positive cells reached the peak at 1 h, and then, the positive cells gradually decreased, while UfSP2 foci positive cell achieved the highest at 2 h (Fig. 2b), suggesting delayed kinetics of UfSP2 recruitment compared to UFL1. To further confirm this result, we utilized the ISce-1 DSB induction system, in which TA-induced ISce-1 translocalizes into the nucleus and cut the only ISce-1 recognition site in the genome DNA, and forms one DSB. We monitored the UfSP2 foci formation after 1 h TA treatment and followed by washout. We detected γH2AX foci were detected at the time of washout, but not UfSP2 foci (Fig. 2c); however, we observed UfSP2 foci, colocalizing with γH2AX foci 30 min after TA washout and then both protein foci diminished 1 h after TA washout (Fig. 2c). UFL1 foci was also detected at the time of TA washout.  Thirty minutes after TA washout, UfSP2 foci colocalized with UFL1 Foci, and both protein foci disappeared 1 h after washout (Fig. 2d). To further confirm that UfSP2 recognizes and removes ufmylation at DSB, we examined both UfSP2 and UFM1 foci following TA washout. Similar to UFL1 foci, UFM1 foci were observed at the time of washout. Thirty minutes after TA washout, UfSP2 foci appeared and UFM1 foci signal became weaker. One hour following TA washout, the signal of both protein foci faded away (Fig. 2e). These results indicate that UfSP2 is recruited with slower kinetics to DSB than UFL1 and removes UFM1 at DSB.

### UfSP2 interacts with MRN complex

**Fig. 3:**
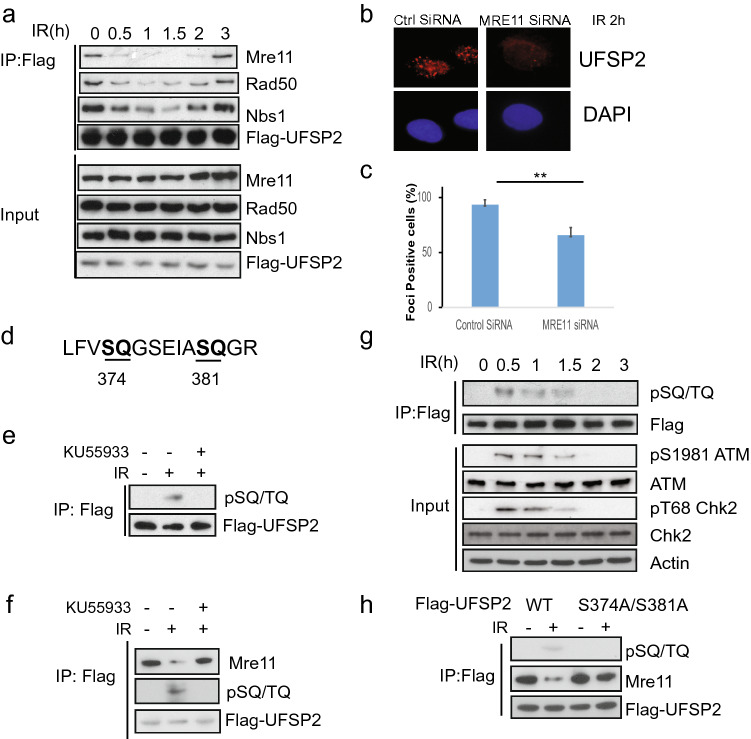
The interaction of UfSP2 with the MRN complex is regulated by ATM-mediated phosphorylation. **a** 293 T cells were transfected with Flag-UfSP2 and treated with 2 Gy IR. Then, the cells were harvested and lysed at indicated time points. The supernatant was incubated with Flag antibodies. The immunoprecipitates were blotted with indicated antibodies. **b**, **c** Immunostaining and quantification of UfSP2 foci in Control siRNA and WIP1 siRNA-transfected cells. **d** Analysis of ATM/ATR substrate motif in UfSP2 protein. **e** Detection of phosphorylated ATM/ATR substrate motif in irradiated cells in the absence or presence of ATM inhibitor KU55933. **f** Dynamic change of phosphorylated UfSP2 mediated by ATM following irradiation. **g** Detection of UfSP2–Mre11 interaction and ATM-induced phosphorylation of UfSP2 in the cells untreated or treated with IR or IR + KU55933. **h** Flag immunoprecipitates from wild-type or S374A/S381A mutant expressing cell lysates were blotted with indicated antibodies

The results above suggest that UfSP2 is recruited to DSB, but the mechanism of this recruitment is still unknown. To address this question, we screened the potential interactor in DNA damage response and found that UfSP2 could interact with Mre11 (Fig. 3a). Accordingly, we also detected interaction between UfSP2 and the other two subunits of the MRN complex: NBS1and Rad50 (Fig. 3a). To better understand the interaction between UfSP2 and MRN complex, we monitor the dynamic change of this interaction following IR. We found that UfSP2 dissociated from MRN promptly following IR, and its interaction with MRN complex was gradually restored at 2 h after IR (Fig. 3a) when ATM activation process is completed, which is consistent with UfSP2 dynamic foci change. Furthermore, depletion of MRE11 impaired UfSP2 foci formation slightly (Fig. 3b, c). These results suggest that the MRN complex is responsible for the recruitment of UfSP2 to the DSBs.

### Phosphorylation of UfSP2 modulates its interaction with the MRN complex

ATM is the important upstream kinase in DSB-induced DNA damage response. Its activity is greatly enhanced shortly following IR and gradually decreased. It is possible that the UfSP2 dissociation from the MRN complex is mediated by ATM-induced phosphorylation. To test this hypothesis, we first analyzed the protein sequence of UfSP2 and found two potential SQ sites (Fig. 3d). IR-induced phosphorylation of UfSP2 was supported by the blot with ATM/ATR phosphorylated substrate antibody (Fig. 3e), and ATM inhibitor KU55933 suppressed this phosphorylation, suggesting that UfSP2 is the substrate of ATM. We further detected the dynamics of ATM-mediated UfSP2 phosphorylation and found that the signal of phosphorylated UfSP2 was abruptly increased following IR, and decreased gradually, displaying similar pattern to the ATM activation and an adverse pattern of UfSP2 association with MRN complex (Fig. 3a, g), suggesting that phosphorylation of UfSP2 by ATM determines the interaction between UfSP2 and MRN complex. This is further supported by sustained UfSP2 interaction with the MRN complex even after DSBs occurred in presence of KU55933 (Fig. 3f). Mutation of both two potential TQ sites in UfSP2 abolished IR-induced phosphorylation and sustained the interaction between UFSP and MRN complex (Fig. 3h). These results indicate that ATM regulates UfSP2 association with MRN complex.

### The phosphatase Wip1 dephosphorylates UfSP2

**Fig. 4:**
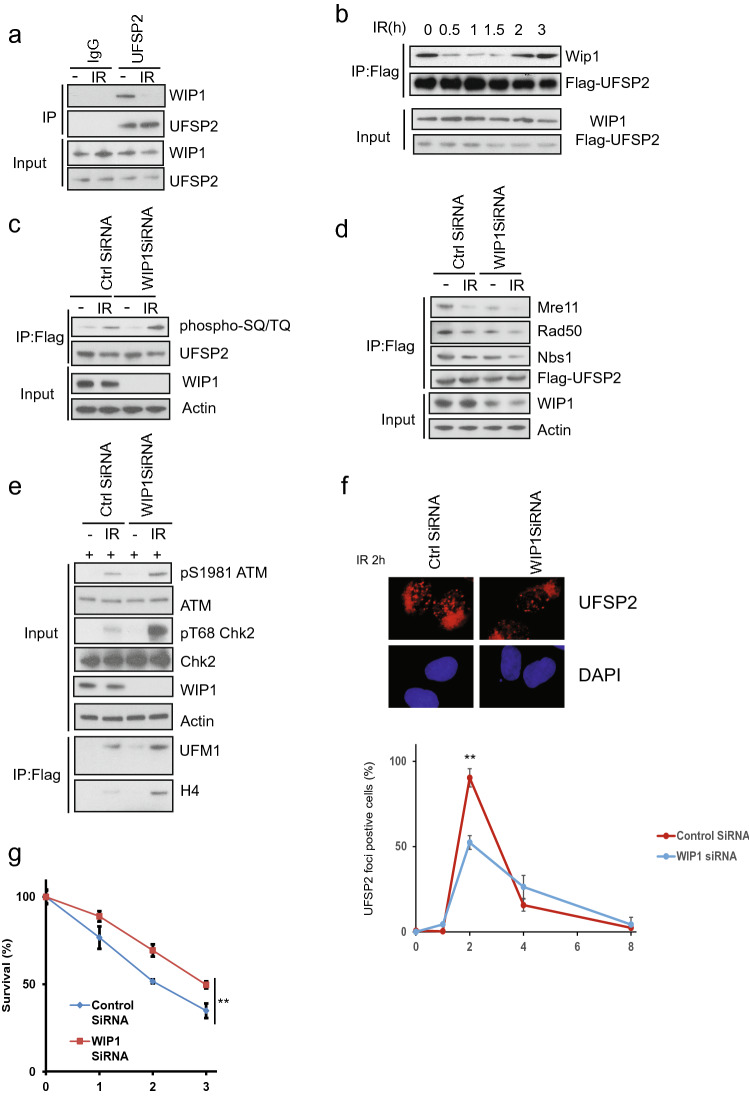
The phosphatase WIP1 removes phospho-group from UfSP2 induced by ATM. **a** Examination of WIP1 interaction with UfSP2 with or without IR. **b** Dynamic change of UfSP2-WIP1 interaction following irradiation. **c**, **d** Detection of ATM-mediated UfSP2 phosphorylation (**c**) and UfSP2 interaction with MRN complex (**d**) in Control siRNA and WIP1 siRNA-transfected cells. **e** Immunoblotting of ATM signaling pathway in Control siRNA and WIP1 siRNA-transfected cells. **f** Immunostaining and quantification of UfSP2 foci in Control siRNA and WIP1 siRNA-transfected cells. g Survival curve of UfSP2 knockout cells transfected with control siRNA and WIP1 siRNA which are treated with indicated doses of IR

As shown in Fig. 3, the UfSP2 dissociation from the MRN complex is mediated by ATM-induced phosphorylation. This event was reversed at the later time point mediated. We hypothesized that this is due to dephosphorylation through an unknown phosphatase during late DNA damage response. Until now, five phosphatases, PP1, PP2A, PP4, PP6, and WIP1, are reported to regulate dephosphorylation events following IR (Freeman & Monteiro, 2010). To determine which phosphatase dephosphorylates UfSP2, we performed immunoprecipitation and found only WIP1 interacted with UfSP2 (Fig. 4a and data not shown). We also studied the dynamic change of interaction between UfSP2 and WIP1 and discovered that IR-induced dissociation of WIP1 from UfSP2 following IR (Fig. 4b). At a later timepoint, this interaction was enhanced, and this pattern was opposite to ATM-mediated phosphorylation of UfSP2 (Fig. 3a). To confirm that WIP1 is the phosphatase responsible for UfSP2 phosphorylation, we knocked down WIP1 with SiRNA and found suppression of WIP1 enhanced IR-induced phosphorylation of UfSP2 (Fig. 4c). Due to the importance of phosphorylation to the interaction between UfSP2 and MRN complex, we also examine this interaction in WIP1 knockdown cells and found UfSP2 interaction with the MRN complex was decreased in WIP1 knockdown cells (Fig. 4d). ATM activation was enhanced in WIP1 knockdown cells (Fig. 4e), and knockdown of WIP1 suppress UfSP2 foci formation at 2 h (Fig. 4f). Consistently depletion of WIP1 induces resistance to IR (Fig. 4g). These results suggest that WIP1 dephosphorylates UfSP2 and enhances the MRN complex-mediated UfSP2 recruitment to DSB.

## Discussion

It is critical to maintain genomic integrity for organism survival. Genotoxic stress induces DNA breaks and leads to malignant transformation. Swift DNA damage response is required to fix the DNA breaks and prevent genome instability. Posttranslational modifications play an important role in DNA damage response and DNA repair. Ubiquitination is one of the well-studied modifications in DSB-induced DNA damage response. When the double-strand break happens, RNF8 binds to phosphorylated MDC1 and RNF8–UBC13 mediates K63-linked polyubiquitylation of L3MBTL2 (Kalb et al., 2014; Mattiroli et al., 2012; Nowsheen et al., 2018; Wang et al., 2004). Ubiquitinated L3MBTL2 is directly recognized by RNF168. RNF168 and then induces the ubiquitylation of H2A-type histones at K13 and K15, which recruits p53-binding protein 1 (53BP1), RAP80, RAD18 and RNF169, and their associated factors, and promotes DNA repair (Botuyan et al., 2006; Chapman et al., 2012a, 2012b; Fradet-Turcotte et al., 2013; Pei et al., 2011).

Previously, we discovered a new modification-ufmylation is involved in ATM activation and DNA damage response. Depletion of any subunit in ufmylation pathway inhibited ufmylation of H4 and suppressed ATM activation (Qin et al., 2019). Consistently, here, we find that loss of UfSP2, the deufmylase, induced enhanced ufmylation of H4, and ATM activation.

ATM activation relies on the positive-feedback signaling to amplify DNA damage response signals. ATM phosphorylates H2AX at Ser139, which is recognized by MDC1 (Lou et al., 2006; Stucki et al., 2005). MDC1 is constitutively phosphorylated (by casein kinase 2) and activates a positive-feedback loop by recruiting more MRN complexes to the damaged chromatin and activate ATM (Chapman et al., 2012a, b; Jungmichel et al., 2012; Liu et al., 2012; Lou et al., 2006, Melander et al., 2008; Spycher et al., 2008; Wang et al., 2019; Wu et al., 2008). Meanwhile, ATM phosphorylates UFL1 at Ser462, and enhances its ufmylase activity. More H4 protein is ufmylated and more SUV39h1 is recruited to the damage site, trimethylating H3 at lysine9, recruiting Tip60 protein, and leading to more ATM kinase activation (Qin et al., 2019). In this study, we found that UfSP2 interacts with the MRN complex, and following DNA damage ATM-mediated phosphorylation disrupts this interaction, and UfSP2 is released from the DSB site. Instead, UFL1 is recruited and activates ATM activation,

On the other hand, ATM deactivation is required for restricting the DNA damage signaling in the region closed to DSB sites. This process is controlled by a series of negative regulators, including protein phosphatases. PP2A is the first phosphatase to be found to negative regulation ATM phosphorylation (Goodarzi et al., 2004). WIP1 is another phosphatase responsible for dephosphorylating ATM and inhibiting its activity (Shreeram et al., 2006). WIP1 knockout MEF cells display increased ATM phosphorylation at Ser 1987 (human Ser 1981) (Darlington et al., 2012). In this study, we find that WIP1 also dephosphorylates UfSP2. Consequently, dephosphorylated UfSP2 bind to the MRN complex again, resulting in deufmylating H4 and disrupting the ATM activation loop. This may restrain excessive ATM activation to prevent cell apoptosis. The restoration of UfSP2 and MRN interaction at 2 h after IR suggests that ATM activation process is completed. While DNA repair process lags behind ATM activation and can last for 8–24 h, so restored interaction between UfSP2 and MRN complex does not indicate the completion of DNA repair.

Together, we discover a rewiring mechanism for the regulation of ATM activity. We suggest that the balance between UFL1 and UfSP2 recruitment and activity is important for proper ATM activation and the DDR. This balancing act will also be important to determine tumor response to radiotherapy and DNA damage-inducing chemotherapy.

## Material and methods

### Cells and reagents

U2OS (TA-induced system) (kindly provided by Dr. Xiaochun Yu) and HEK 293 T (ATCC) cells were cultured in DMEM supplemented with 10% FBS. All cell lines were kept in a humidified 37 °C 5% CO_2_ 5% O_2_ incubator. The cells were irradiated with 0.5 Gy for immunofluorescence studies and 2 Gy for western blot/co-immunoprecipitation assays. UfSP2-Flag, UfSP2 (C302S), and His-UFM1 plasmids were described previously. Triamcinolone acetonide, and the ATM inhibitor KU55933 (Sigma) were used in this study. WIP1 siRNA was purchased from Santa Cruz.

Anti-NBS1 antibody (A301-284A, 1:1000 for western) was purchased from Bethyl. Anti-actin (A5316, 1:10,000) was purchased from Sigma. Anti-ATM (2873, 1:1000 for western), anti-pSer1981 ATM (13050, 1:1000 for western), anti-Mre11 (4847, 1:1000 for western), anti-Rad50 (3427, 1:1000 for western), anti-SQ/TQ motif (9607, 1:1000 for western), anti-Chk2 (2662, 1:1000 for western), and anti-phosphoChk2 (2197, 1:1000 for western) antibodies were purchased from Cell Signaling. Anti-γH2AX (05-636, 1:1000 for IF) and anti-MDC1 (05-172, 1:1000) antibodies were purchased from Millipore. Anti-UfSP2 rabbit antibody for foci detection was purchase from Santa Cruz. For immunoprecipitation assay, Anti-IgG, Light Chain Specific antibodies were used (Jackson immunoresearch).

Lipofectamine 2000 Transfection Reagent (Invitrogen), RNAMAX (Invitrogen), and Mirus TransIT Transfection Reagent (Mirus Bio LLC) were used for carrying out transfections following the manufacturer’s protocols.

### Western blot and immunoprecipitation

NETN buffer (20 mM Tris–HCl, pH 8.0, 100 mM NaCl, 1 mM EDTA, 0.5% Nonidet P-40, 50 mM β-glycerophosphate, 10 mM NaF, and 1 mg per ml each of pepstatin A and aprotinin) was used for lysing cells. The cell lysates were spun, and the supernatant indicated antibodies, and 20 µl protein A or protein G Sepharose beads (Amersham Biosciences) for 4 h or overnight at 4 °C. The immunoprecipitates were washed with ice cold NETN buffer and then incubated with 1× Laemmli buffer for boiling. The samples were analyzed by SDS-PAGE. Samples in SDS-PAGE gels were then transferred to PVDF membrane with semi-dry method (Trans-Blot^®^ Turbo™ Transfer System, Bio-Rad). Following incubation with 5% milk, primary antibodies, secondary antibodies, and ECL, the western blot signals were detected by X-ray films.

### Flag-His-UFM1 purification

The assay was performed as describe before. Ufm1ΔC2-Flag-His were cotransfected with other plasmids and irradiated. Then, the cells were lysed with denaturing buffer. After sonication, the supernatant was incubated with nickel bead for 2 h. The nickel beads were washed three times, and then, the His-tagged protein was eluted off with elution buffer. The purified proteins were further dialyzed with Amico ultra-spin column and then incubated with Flag agarose beads for 1 h. The binding protein was eluted with Flag peptide.

### Colony formation assay

The cells were seeded in each well of 6-well plates. Sixteen hours later, cells were treated with ionizing radiation with indicated dose, and incubated for further 10–14 days at 37 °C. Colonies were stained with methylene blue and counted.

### Inducible single DSB system

U2OS cells with RFP-I-SceI-GR stable expression were incubated with the synthetic glucocorticoid (GR) ligand triamcinolone acetonide (TA purchased from Sigma) at the final concentration of 0.1 µM to induce the translocation of RFP-I-SceI-GR from cytoplasm into nucleus. Pictures were recorded by Nikon eclipse 80i fluorescence microscope.

### Statistical analysis

Data in this study were shown as mean ± SD or mean ± sem of at least three independent experiments. Comparisons were carried out with a two-tailed unpaired Student’s t test and two-way or one-way ANOVA using graph pad prism (∗*p* < 0.05, ∗∗*p* < 0.01).
